# Deletion of Lipoteichoic Acid Synthase Impacts Expression of Genes Encoding Cell Surface Proteins in *Lactobacillus acidophilus*

**DOI:** 10.3389/fmicb.2017.00553

**Published:** 2017-04-11

**Authors:** Kurt Selle, Yong J. Goh, Brant R. Johnson, Sarah O’Flaherty, Joakim M. Andersen, Rodolphe Barrangou, Todd R. Klaenhammer

**Affiliations:** ^1^Functional Genomics Graduate Program, North Carolina State UniversityRaleigh, NC, USA; ^2^Department of Food, Bioprocessing and Nutrition Sciences, North Carolina State UniversityRaleigh, NC, USA; ^3^Microbiology Graduate Program, North Carolina State UniversityRaleigh, NC, USA

**Keywords:** lipoteichoic acid, *Lactobacillus acidophilus*, probiotic, S-layer, cell surface, proteomics, transcriptomics

## Abstract

*Lactobacillus acidophilus* NCFM is a well-characterized probiotic microorganism, supported by a decade of genomic and functional phenotypic investigations. *L. acidophilus* deficient in lipoteichoic acid (LTA), a major immunostimulant in Gram-positive bacteria, has been shown to shift immune system responses in animal disease models. However, the pleiotropic effects of removing LTA from the cell surface in lactobacilli are unknown. In this study, we surveyed the global transcriptional and extracellular protein profiles of two strains of *L. acidophilus* deficient in LTA. Twenty-four differentially expressed genes specific to the LTA-deficient strains were identified, including a predicted heavy metal resistance operon and several putative peptidoglycan hydrolases. Cell morphology and manganese sensitivity phenotypes were assessed in relation to the putative functions of differentially expressed genes. LTA-deficient *L. acidophilus* exhibited elongated cellular morphology and their growth was severely inhibited by elevated manganese concentrations. Exoproteomic surveys revealed distinct changes in the composition and relative abundances of several extracellular proteins and showed a bias of intracellular proteins in LTA-deficient strains of *L. acidophilus*. Taken together, these results elucidate the impact of *ltaS* deletion on the transcriptome and extracellular proteins of *L. acidophilus*, suggesting roles of LTA in cell morphology and ion homeostasis as a structural component of the Gram positive cell wall.

## Introduction

*Lactobacillus acidophilus* NCFM is a widely consumed probiotic strain that has been at the forefront of genomic characterization of probiotic functionality (Sanders and Klaenhammer, 2001; Altermann et al., 2005). Probiotics are live microorganisms, which when administered in adequate amounts, confer a health benefit to the host (Hill et al., 2014). Recently, emphasis has been placed on elucidating the molecular mechanisms and regulatory networks responsible for probiotic activity (Bron et al., 2011). Unraveling probiotic mechanisms has been greatly enabled by genome sequencing (Kleerebezem et al., 2003; Pridmore et al., 2004; Makarova et al., 2006) development of genetic tools (Leenhouts et al., 1991; Trieu-Cuot et al., 1991; Law et al., 1995; Maguin et al., 1996; Russell and Klaenhammer, 2001; Goh et al., 2009), transcriptomics (Azcarate-Peril et al., 2005; Denou et al., 2007; Marco et al., 2010), and increasingly, proteomics (Kelly et al., 2005; Izquierdo et al., 2009; Johnson et al., 2013, 2016). Many of these approaches have been extensively applied to investigate cell-surface constituents, as they are highly strain-specific and are uniquely positioned to effect host–microbe crosstalk (Buck et al., 2005; Kleerebezem et al., 2010). Predictive and functional genomics studies of *L. acidophilus* NCFM have revealed that surface layer (Slps) and surface layer associated proteins (SLAPs) (Buck et al., 2005; Johnson et al., 2013), sortase-dependent proteins (Call et al., 2015), and adhesion exoproteins mediate adhesion and immunomodulatory activities of *L. acidophilus* (Buck et al., 2005). *L. acidophilus* synthesizes three Slps, the dominant SlpA and minor components SlpB and SlpX (Goh et al., 2009). As the major component of the S-layer in *L. acidophilus*, SlpA contributes to interaction with intestinal epithelial cells and to modulation of immune responses (Buck et al., 2005; Konstantinov et al., 2008; Lightfoot et al., 2015).

Lipoteichoic acid (LTA) is a large amphiphilic interfacial polymer consisting of a poly-glycerol or ribitol-phosphate backbone attached to a glycolipid moiety with a hydrocarbon tail embedded in the cell membrane (Weidenmaier and Peschel, 2008). LTA serves pleiotropic roles in Gram-positive physiology and is a major immunomodulatory cell surface component that typically elicits an inflammatory response from antigen-presenting cells (Ginsburg, 2002). Genetic modification or removal of LTA from probiotic microbes abrogates the expression of pro-inflammatory cytokines and elicits expression of regulatory cytokines, conferring an increased capacity to alleviate mucosal inflammation (Grangette et al., 2005; Mohamadzadeh et al., 2011). Indeed, disruption of the gene encoding lipoteichoic acid synthase (*ltaS*) causes a shift in the immune system interaction of *L. acidophilus* toward an IL-10 dependent regulatory phenotype, which has been characterized using murine disease models of colitis and colonic polyposis *in vivo* (Mohamadzadeh et al., 2011; Khazaie et al., 2012). To assign immunomodulatory properties specific to SlpA, a *L. acidophilus* derivative deficient in LTA and minor S-layer components SlpB and SlpX was created (Lightfoot et al., 2015). Administration of live cells of the strain mitigated colitis in a murine model and restored epithelial barrier integrity. Of note, purified SlpA from the strain was also sufficient for ameliorating colitis and restoration of epithelial barrier integrity (Lightfoot et al., 2015).

*LtaS* knockout mutations are often lethal to bacteria in a phylogenetically dependent manner (Gründling and Schneewind, 2007). Consequently, few studies detail successful creation and characterization of LTA-deficient strains in either pathogenic or beneficial Gram-positive bacteria (Schirner et al., 2009; Webb et al., 2009). These foundational studies established localization and interaction of LTA with cell division machinery, providing important insights into the functions of LTA in the cell wall. Despite the creation and immunological characterization of LTA-deficient strains of *L. acidophilus*, little is known concerning the overlap and potential redundancy of cell-surface molecules contributing toward fundamental bacterial physiology. We investigated two *L. acidophilus* mutants deficient in LTA, NCK2025 (Δ*ltaS*) and NCK2187 (Δ*upp*Δ*slpB* Δ*slpX*Δ*ltaS*) to assess the compensatory transcriptional and extracellular protein changes caused by deletion of LTA. The transcriptome and exoproteome screening suggested that LTA impacts cell morphology and ion homeostasis in *L. acidophilus*, which was supported by preliminary phenotypic analysis of cellular morphology and growth in high manganese conditions.

## Materials and Methods

### Bacterial Strains

All bacterial strains and plasmids are listed in **Table 1**. Bacterial cultures were cryopreserved in their respective media with a 15% glycerol concentration (vol/vol) and stored at -80°C. *L. acidophilus* was propagated in de Mann, Rogosa and Sharpe (MRS) (Difco Laboratories, Inc., Detroit, MI, USA) broth under static aerobic conditions at 37°C, or on MRS agar (1.5% wt/vol agar, Difco) incubated anaerobically at 37°C for 48 h. Concentrations of 2 μg/mL of erythromycin (Em) (Sigma-Aldrich, St. Louis, MO, USA) and 2–5 μg/mL of chloramphenicol (Cm) (Sigma) were used for plasmid selection in *L. acidophilus* NCFM, when appropriate. Selection for 5-fluorouracil resistant *L. acidophilus* was performed by supplementing glucose semi-defined (GSDM) (Kimmel and Roberts, 1998) agar with a final concentration of 100 μg/mL of 5- fluorouracil (Sigma) (Goh et al., 2009). *Escherichia coli* EC1000 was propagated aerobically in Luria-Bertani (Difco) broth at 37°C, or on brain-heart infusion (Difco) solid medium supplemented with 1.5% agar. Antibiotic selection of *E. coli* was maintained with 40 μg/mL kanamycin (Kn) and 150 μg/mL of Em for recombinant *E. coli*, when appropriate.

**Table 1:** Bacterial strains and plasmids.

	Characteristics	Source or reference
**Strains**		
*L. acidophilus*		
NCK1909	NCFM carrying a 315-bp in-frame deletion within the *upp* gene	Goh et al., 2009
NCK1910	NCK1909 harboring pTRK669; host for pORI-based counterselective integration vector	Goh et al., 2009
NCK1964	NCK1909 carrying a 1,170-bp in-frame deletion in the *slpB* gene	Goh and Klaenhammer, unpublished
NCK2025	NCFM carrying a 2,022-bp in-frame deletion in the *ltaS* gene	Mohamadzadeh et al., 2011
NCK2030	NCK1964 carrying a 1,356-bp in-frame deletion in the *slpX* gene	Goh and Klaenhammer, unpublished
NCK2187	NCK2030 carrying a 2,022-bp in-frame deletion in the *ltaS* gene	Lightfoot et al., 2015
*E. coli*		
EC1000	RepA^+^ JM101; Km^r^; *repA* from pWV01 integrated in chromosome; host for pORI-based plasmids	Law et al., 1995
**Plasmids**		
pTRK935	3.0 kb; pORI28 with a *upp* expression cassette and the *lacZ’* from pUC19 cloned into BglII/XbaI sites; serves as counterselective integration vector	Goh et al., 2009
pTRK956	4.5 kb; pTRK935 with a mutated copy of *slpX* cloned into BamHI/SacI sites	Goh et al., 2009
pTRK957	4.5 kb; pTRK935 with a mutated copy of *slpB* cloned into BamHI/SacI sites	Goh and Klaenhammer, unpublished
pTRK1052	4.5 kb; pTRK935 with a mutated copy of *ltaS* cloned into BamHI/SacI sites	Lightfoot et al., 2015


### DNA Isolation, Manipulation, and Transformation

All kits, enzymes, and reagents were used according to the manufacturers’ instructions. DNA purification and cloning were performed as previously described (Goh et al., 2009). Purification of genomic DNA from *L. acidophilus* employed a ZR Fungal/Bacterial MiniPrep kit (Zymo Research, Corp., Irvine, CA, USA). Plasmid DNA was isolated from *E. coli* using Qiagen Spin miniprep kit (Qiagen, Inc., Valencia, CA, USA). High fidelity PCR amplification of DNA was performed with PFU HS II DNA polymerase (Stratagene, Corp., La Jolla, CA, USA). Routine PCRs were conducted with Choice-*Taq* Blue polymerase (Denville Scientific, Inc., Meutchen, NJ, USA). Primers for PCR amplification were purchased from Integrated DNA Technologies and are listed in Supplemental Table 1 (Coralville, IA, USA). DNA amplicons were separated using 0.8% agarose gel electrophoresis and stained with ethidium bromide for visualization. DNA extraction from agarose gels was performed with a Zymoclean DNA gel recovery kit (Zymo Research). Restriction endonucleases were acquired from Roche Molecular Biochemicals (Indianapolis, IN, USA). Ligations were performed with New England Biolabs (Beverly, MA, USA) quick T4 ligase. Sequencing was performed by Davis Sequencing Inc. (Davis, CA, USA). Rubidium chloride competent *E. coli* cells were prepared as previously described and frozen at -80°C (Hanahan, 1983). Heat shock transformants of the ligation mixture were subsequently screened by PCR for inserts using primers flanking the multiple cloning site. Plasmids putatively containing inserts were sequenced across the multiple cloning site to ensure fidelity. Newly constructed integration plasmids were electroporated into competent cells containing the temperature-sensitive helper plasmid, pTRK669, according to methods described previously (Russell and Klaenhammer, 2001). Penicillin G at a concentration of 10 μg/mL was employed in the preparation of the competent cells to promote electroporation efficiency (Wei et al., 1995).

### Microscopy and Growth Curves

Cultures were propagated from frozen stocks and subcultured twice at a 1% (vol/vol) inoculum in MRS under standard conditions and harvested after 16 h incubation (stationary phase). Cell morphology and chain length was visualized using a Nikon Eclipse E600 phase contrast microscope with a Q-Imaging Micropublisher Camera attachment. At least 40 cells from each of three biological replicates were measured using Image Pro Insight software (Media Cybernetics, Inc., Rockville, MD, USA). Chain lengths were averaged and means were compared using a two-tailed *t*-test with unequal variance, at a significance threshold of *p* < 0.05.

For comparing the growth profiles of each *L. acidophilus* derivative, cultures were propagated from frozen stocks in MRS and subcultured twice at a 1% (vol/vol) inoculum in GSDM under standard conditions. The growth of each strain in both standard and high Mn medium was monitored in triplicate using a Spec-20 spectrophotometer at 600 nm and reported as an average.

### Transcriptional Analysis

RNA was extracted from log phase cultures (optical density of 0.6–0.8) grown in MRS medium under static conditions at 37°C. Cells were harvested by centrifugation 4000 × *g* for 10 min and then flash frozen using an ethanol-dry ice bath and stored at -80°C. For RNA isolation, each frozen pellet was resuspended in 1 mL of Tri-reagent (Zymo Direct-zol RNA MiniPrep Kit -Zymo Research, Irvine, CA, USA) and cell lysis was performed using a Mini bead beater set to homogenize for five cycles of 1 min beating alternated with 1 min incubation on ice. Total RNA was isolated according to protocols from TRI-reagent and the Direct-zol RNA MiniPrep Kit. Each RNA preparation was quantified with NanoDrop and analyzed for quality using an Agilent 2100 Bioanalyzer (Agilent Technologies, Santa Clara, CA, USA). Preparation of each mRNA library and RNA-sequencing were performed at the High-Throughput Sequencing and Genotyping Unit of the Roy J. Carver Biotechnology Center, University of Illinois at Urbana-Champaign. For each sample, ribosomal RNA was removed with the Ribozero Bacteria Kit (Illumina, San Diego, CA, USA) followed by library preparation with the TruSeq Stranded RNA Sample Prep Kit (Illumina, San Diego, CA, USA). Single-read RNA-sequencing was performed using an Illumina HiSeq 2500 Ultra-High-Throughput Sequencing system (Illumina, San Diego, CA, USA) with a read length of 180 nucleotides. Raw sequencing reads were quality assessed using FastQC Version 0.11.3^1^ and processed using Geneious 8.0.5 (Kearse et al., 2012). Briefly, after adaptor sequences were removed, raw reads were quality trimmed to remove bases with an error probability limit of 0.001 (Phred score of 30) and filtered to remove reads shorter than 20 nt. These quality trimmed and filtered sequences were then mapped to the reference genomes with default settings within Geneious 8.1.7. Two different approaches were employed to assess the RNA-seq data; differential expression (DE)-seq, which uses median of gene expression ratios normalization, and two-way hierarchical clustering using the trimmed mean of M-values normalization method. Expression was calculated in Geneious and compared using the median of gene expression ratios method, also known as DE-Seq (Dillies et al., 2012). The cutoff thresholds for DE of genes were a minimum of a twofold change and a *p*-value < 1.36 × 10^-5^, adjusted by the Bonferroni correction for multiple testing, treating each ORF as an independent test. LTA positive strains were compared with LTA negative strains in a pairwise fashion. JMP genomics (Statistical Analysis Software, Cary, NC, USA) was used to construct two-way hierarchical clustering heat maps with centered rows under the fast-ward algorithm using the log_2_ transformed trimmed mean of M-values normalization method.

### Extraction and Identification of Extracellular, Non-covalently Bound Cell Surface Proteins

Non-covalently bound cell surface proteins, including S-layer proteins (SLPs) and S-layer associated proteins (SLAPs) were extracted from the *Lactobacillus* strains using LiCl denaturing salt, as described previously (Johnson et al., 2013). SLP and SLAP pellets were resuspended in 10% (w/v) SDS (Fisher, Waltham, MA, USA). Proteins were quantified via bicinchoninic acid assay kit (Thermo Scientific, Waltham, MA, USA) and 10 ng was loaded and visualized via SDS-PAGE using precast 4–20% Precise Tris-HEPES protein gels (Thermo Scientific, Waltham, MA, USA). Gels were stained using AcquaStain (Bulldog Bio, Portsmouth, NH, USA) according to the instructions from the manufacturer.

Surface layer associated proteins extracted from the various *L. acidophilus* strains were identified using LC–MS/MS from the Genome Center Proteomics Core at the University of California, Davis, as described previously (Johnson et al., 2013). For all analyses, total spectral counts were utilized as a semi-quantitative indicator of protein abundance (Liu et al., 2004). Two-way clustering of total spectral counts was performed using JMP Genomics (version 5, SAS). Protein domains were identified for analysis using the Pfam protein family database (Finn et al., 2014).

## Results

### LTA-Deficiency Elicits Compensatory Transcriptional Changes in *L. acidophilus*

**FIGURE 1:**
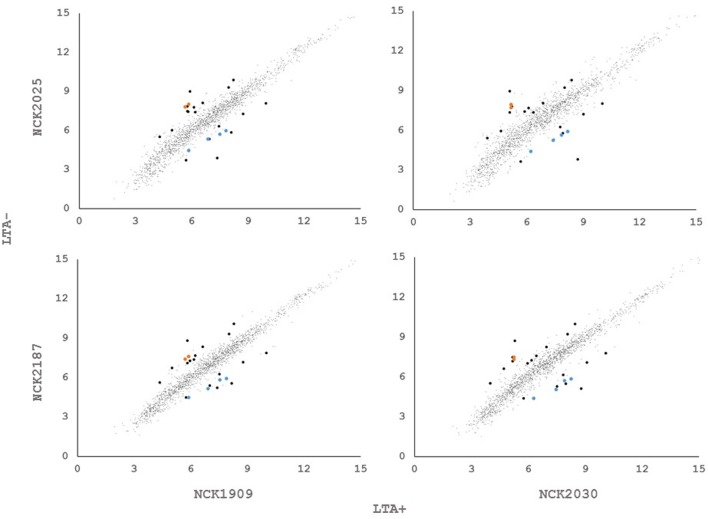
**XY plots of log2 transformed, normalized transcripts per kilobase million (TPM) metric of open reading frames in *Lactobacillus acidophilus*.** Black data points meet the significance thresholds *p* < 1.36e^-5^, α = 0.05) and fold change (>2) in each pairwise comparison. Colored data points indicate differentially expressed genes in operons.

**FIGURE 2:**
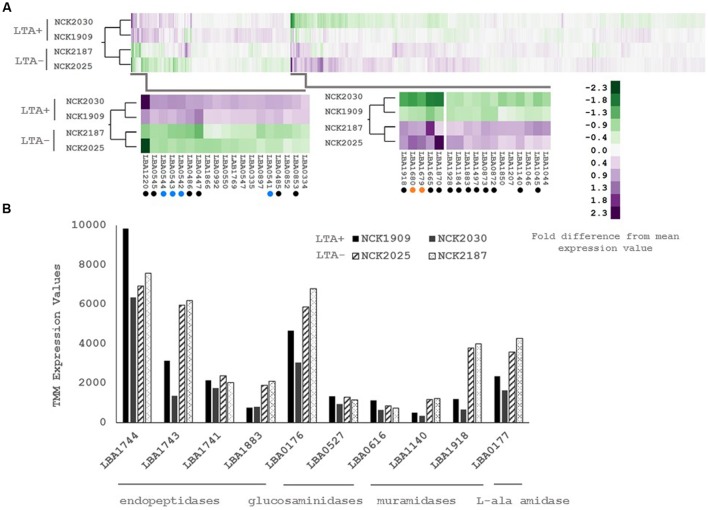
**(A)** RNA-seq transcriptional profiles of *L. acidophilus* derivatives visualized by two-way hierarchical clustering of trimmed mean of M-values (TMM) normalized reads per gene. Black circles denote differentially expressed genes and colored circles denote differentially expressed operons. **(B)** RNA-seq transcriptional profiles of predicted peptidoglycan turnover proteins in *L. acidophilus.* Values are TMM gene expression levels. Non-solid bars indicate LTA-deficient strains.

In order to determine whether loss of LTA caused compensatory transcriptional changes, and to identify genes relevant to the function of LTA in *L. acidophilus*, we used RNA-seq to characterize the global transcriptional profile of LTA mutants. RNA sequencing statistics for each strain were summarized in Supplemental Table 2. Differentially expressed genes specific to LTA deletion were identified by pairwise comparison of each LTA^+^ strain (NCK1909 and NCK2030) with each LTA^-^ strain (NCK2025 and NCK2187). Genes meeting the cutoffs for DE in all four comparisons were considered differentially expressed due to absence of LTA. Each pairwise comparison was visualized as an XY plot of log_2_ transformed normalized transcripts per million, with differentially expressed genes marked by enlarged circles (**Figure 1**). Using this conservative approach, a total number of 24 genes were found to be differentially expressed across all pairwise comparisons (**Table 2**), 10 of which were encoded in putative operons. Overall, for differentially expressed genes encoded in putative operons, co-encoded genes followed the same trend despite not being significantly differentially expressed (Supplemental Table 3). In contrast, *ltaS* (LBA0447) was downregulated due to the in-frame DNA deletion, but expression for the rest of the LTA biosynthetic operon was unaffected. Two-way hierarchical clustering was also used to correlate expression patterns with the LTA genotypes (**Figure 2**). Selected portions of the heat map display the genes for which expression most closely correlated with LTA genotype, with differentially expressed genes identified again being denoted with circles.

**Table 2:** List of differentially expressed genes.

Gene	Predicted function	Range of log_2_ change (LTA^-^/LTA^+^)
**Downregulated genes**
LBA0485	Hypothetical protein	-1.5 to -2.0
LBA0486	Hypothehical protein	-1.9 to -2.2
LBA0447	Lipoteichoic acid synthase	-2.1 to -2.8
LBA0541	cadA	-1.3 to -1.8
LBA0542	Heavy metal-transporting ATPase	-1.7 to -2.6
LBA0543	Hypothetical protein	-1.7 to -2.5
LBA0544	Transcriptional regulator	-1.7 to -2.2
LBA0853	*N*-acetyldiaminopimelate deacetylase	-1.1 to -1.7
LBA1220	Pyridine mercuric reductase	-2.2 to -4.9
LBA1801	Hypothetical protein	-1.1 to -2.2
**Upregulated genes**		
LBA0872	Hypothetical protein	1.1 to 1.7
LBA0873	Hypothetical protein	1.3 to 1.8
LBA1045	Glutamine ABC transporter ATP-binding protein	1.1 to 2.0
LBA1140	Lysin	1.2 to 1.5
LBA1184	Hypothetical protein	1.1 to 1.7
LBA1497	Hypothetical protein	1.1 to 1.3
LBA1665	oppA	2.1 to 3.4
LBA1679	ABC transporter permease	1.7 to 2.4
LBA1680	ABC transporter ATP-binding protein	1.7 to 2.8
LBA1690	Hypothetical protein	1.0 to 1.2
LBA1870	Maltose phosphorylase	1.4 to 3.9
LBA1883	NLP-P60 secreted protein	1.1 to 1.5
LBA1918	lysA	1.7 to 2.4
LBA1928	Hypothetical protein	1.0 to 1.8


Three of the upregulated genes corresponded to *N*-acetylmuramidases (LBA1140 and LBA1918) and an endopeptidase (LBA1883), which are predicted to function in peptidoglycan turnover (**Table 2**). To survey how *ltaS* deletion impacted collective expression of peptidoglycan hydrolases, we compared expression values across each predicted peptidoglycan hydrolase gene in *L. acidophilus* (**Figure 2**). Interestingly, an endopeptidase (LBA1743), a *N*-acetylglucosiminidase (LBA0176), and an L-alanine amidase (LBA0177) exhibited trends of increased expression, although they did not meet the threshold for DE. Also striking was the lack of predicted function for 10 of the 24 differentially expressed genes, which are putative hypothetical proteins (**Table 2**).

### LTA Maintains Cell Shape and Provides Resistance to Manganese

**FIGURE 3:**
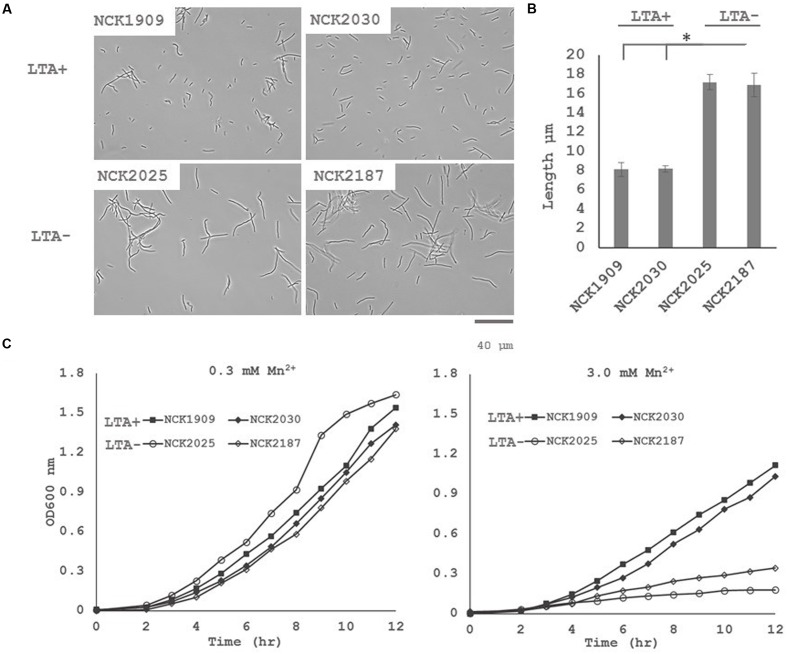
**(A)** Microscopy images of wild-type *L. acidophilus* and LTA derivatives at 400x magnification and **(B)** comparison of stationary phase cultures for average chain lengths. 40 cells from three independent biological replicates were measured and their means compared using a two-tailed, equal variance Student’s *t*-test. Asterisk denotes *p* < 0.05. **(C)** Growth profiles of *L. acidophilus* NCFM derivatives in normal glucose semi-defined medium (left) and high manganese glucose semi-defined medium (right). Data are reported as the average of three biological replicates and open markers indicate LTA-deficient strains.

Differential expression of putative peptidoglycan hydrolases suggested that cell division or cell morphology could have been impacted by *ltaS* deletion. Thus, 40 cells from stationary phase cultures were measured and their average length compared in triplicate (**Figure 3**). *L. acidophilus* strains lacking *ltaS* (NCK2025 and NCK2187) exhibited an elongated cellular morphology, exhibiting a ∼2X increased length compared to the LTA^+^ strains (*p* < 0.05). Qualitatively, many cells of the NCK2025 culture exhibited bending or curving, but this was less apparent in the NCK2187 culture (**Figure 3**). These results are evidence of direct impacts of LTA on cell morphology and that *ltaS* deletion in rod-shaped bacteria promotes cell elongation.

The DE of an operon predicted to function in heavy metal resistance suggested that LTA mutants may have a reduced threshold of ion tolerance. To assess whether LTA plays a similar role in ion homeostasis in *L. acidophilus*, growth profiles in semi-defined medium with standard Mn^2+^ concentrations (0.3 mM) and with elevated Mn^2+^ concentrations (3 mM) were compared (**Figure 3**). Under standard Mn^2+^ concentration, minimal difference in growth between the LTA^+^ and LTA^-^ strains was observed. By contrast, both LTA^-^ strains displayed severe growth inhibition in the high Mn^2+^ concentration when compared to LTA^+^ strains, irrespective of deletion of SlpB or SlpX.

### LTA Mutants Exhibit Altered Cell Surface Protein Profiles

**FIGURE 4:**
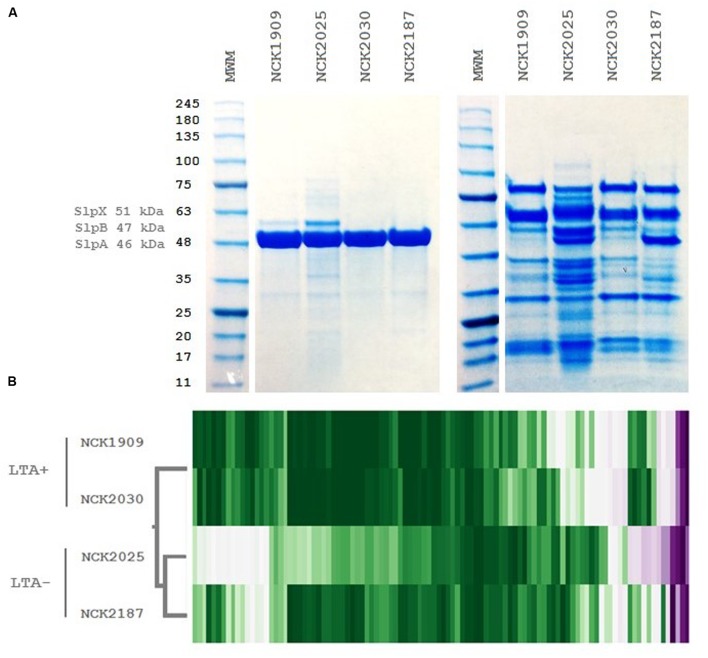
**Extracellular protein profiles of *L. acidophilus* LTA derivatives.** **(A)** SDS-PAGE images of S-layer (left) and S-layer associated protein fractions (right) and **(B)** two-way hierarchical clustering of spectral counts per extracellular protein generated by liquid chromatography tandem mass spectrometry. The molecular weight marker denotes kDa size.

**FIGURE 5:**
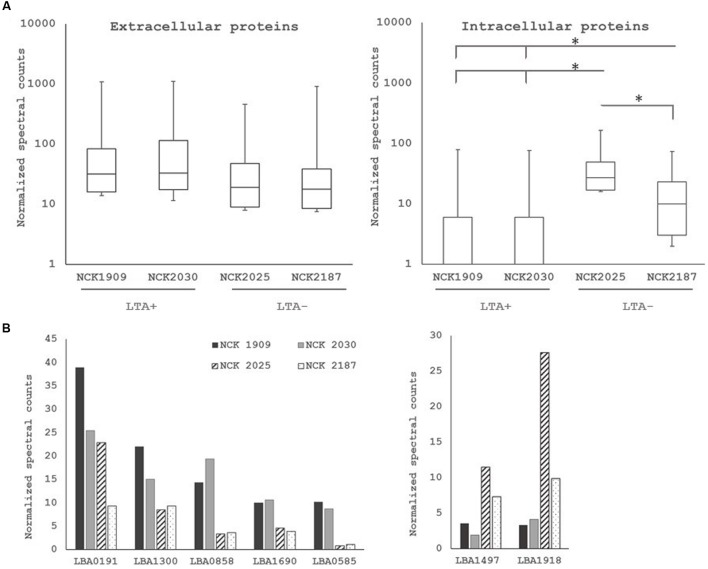
**(A)** Distribution of normalized spectral counts for proteins with (left) and without (right) secretion signals. Asterisk denotes *p* < 0.05. **(B)** Comparison of normalized spectral counts of select extracellular proteins across *L. acidophilus* derivatives. Non-solid bars indicate LTA-deficient strains.

To examine the role of LTA in the presentation of non-covalently attached cell surface proteins, exoproteome screenings of Slps and SLAPs were performed on each *L. acidophilus* strain. Non-covalently bound extracellular proteins, including Slps and SLAPs were isolated from these strains and visualized (**Figure 4**). The SDS-PAGE image reflects the Slp profile expected for each of the strains; specifically that NCK1909 and NCK2025 are SlpA^+^ SlpX^+^ whereas NCK2030 and NCK2187 are SlpA^+^ SlpX^-^. Proteins in the SLAP fraction were identified using LC–MS/MS and analyzed and compared using two-way clustering based on the similarity of the identified proteins (**Figure 4**). It is clear that the SLAP fraction of NCK2025 is distinct from those found in the other strains, including NCK2187. Notably, although both LTA-deficient strains NCK2025 and NCK2187 cluster together, NCK2025 exhibits a distinctly unique profile in both the SDS-PAGE image and the heat map. These data suggest that the absence of LTA alters non-covalently bound extracellular proteins but also indicates a distinction between NCK2025 and NCK2187 in their exoproteome composition. Spectral counts for each SLAP protein were considered a semi-quantitative measure of relative protein abundance and were compared across the LTA^+^ and LTA^-^ strains. To assess differences in intracellular and extracellular protein abundance, the distribution of spectral counts corresponding to proteins with and without a putative secretion signal were compared (**Figure 5**). The LTA^-^ strains exhibited significantly higher distributions of intracellular protein counts relative to the LTA^+^ strains (*p* < 0.05). Moreover, the distribution of intracellular protein counts were significantly higher in NCK2025 than in NCK2187. We then compared spectral counts for selected proteins with secretion signals (**Figure 5**). Five SLAP proteins were present at decreased levels in the LTA-deficient strains, including putative fibronectin-binding protein FbpB (LBA0191), a penicillin-binding protein (LBA0858), and two glycerol-3-phosphate ABC transporters (LBA0585 and LBA1641). Similarly, there were two extracellular proteins with increased levels in LTA-deficient strains including a protein of unknown function (LBA1497) and the LysA muramidase (LBA1918). These results show that removal of LTA from the cell surface increases the presence of intracellular proteins in extracellular preparations, and impacts extracellular protein levels in *L. acidophilus* NCFM.

## Discussion

Genetic tools for functional genomics combined with high-throughput data technologies have contributed to 10 years of unraveling host–microbe crosstalk in *L. acidophilus* NCFM (Altermann et al., 2005). These approaches have led to discovery and characterization of genes involved in probiotic activity and identified cell-surface constituents that positively impact human health. LTA is a classical molecule in host–microbe interactions, the deletion of which has proven to shift immunomodulatory responses toward ameliorating inflammation in models of immunological diseases (Lebeer et al., 2012). However, comprehensive characterization of LTA functions in Firmicutes remains a challenge given the pleiotropy of the *ltaS* gene and difficulty in achieving knockouts. Foundational studies have reported phenotypic characteristics of LTA-deficient bacteria in *Bacillus subtilis*, *Staphylococcus aureus* and *Listeria monocytogenes*, and have provided insights into the intermolecular interactions of LTA in the cell wall (Gründling and Schneewind, 2007; Schirner et al., 2009; Webb et al., 2009). In order to elucidate effects of LTA on the physiology of *L. acidophilus*, we investigated LTA-deficient strains NCK2025 and NCK2187.

To investigate the compensatory transcriptional responses in LTA-deficient *L. acidophilus* mutants, we used RNA-seq to analyze the transcriptional profiles of four strains. While the focus of our analysis was to identify differentially expressed genes correlated with LTA genotype, the vast majority were not significantly altered. We were able to identify 24 genes expression of which was significantly different in the LTA-deficient strains. Although some of the genes identified were related to predicted functions of LTA, their interrelation with LTA was unclear. Some putative peptidoglycan turnover genes in *L. acidophilus* were affected by *ltaS* deletion, especially LysA (LBA1918), a muramidase (LBA1140), and an endopeptidase (LBA1883). There is evidence suggesting that LTA excludes autolysins from delocalized peptidoglycan substrates except for the cell septa (Steen et al., 2003). It may be possible that the delocalized activity of autolysins caused upregulation of peptidoglycan turnover genes to compensate for lack of specific endolysin activity at the cell septa. Some of the genes downregulated in LTA-deficient strains were associated with heavy metal resistance pathways. LBA0541 encodes cadA, a non-specific ATPase efflux protein, LBA0542 also encodes an ATPase efflux protein, and LBA1220 encodes a pyridine mercuric reductase. It is unclear whether DE of these genes is related to or partially responsible for the loss of Mn^2+^ resistance in LTA^-^ strains, but further investigation may reveal or define their intersection with LTA activity.

Our results show *ltaS* deletion promotes cell elongation in *L. acidophilus*, which is likely mediated through aberrant cell division and that LTA-deficient *L. acidophilus* strains are highly sensitive to elevated Mn^2+^ concentrations. We also demonstrate that LTA mutants exhibit DE patterns compared to parent strains. Specifically, we observed 24 genes whose expression significantly correlated with LTA genotypes, 10 of which were without any annotated function. Of those differentially expressed genes with predicted function, several were related to cell wall turnover and to heavy metal resistance. We postulate that differences in expression of these genes may be related to the cell morphology and Mn^2+^ toxicity phenotypes observed, although the direct mechanisms and regulatory networks must be further investigated. We hypothesized that removal of LTA would affect composition of the exoproteome due to its capacity to act as a scaffold for extracellular proteins, and were able to identify proteins whose levels were altered in the SLAP fraction of the LTA^-^ strains. Importantly, the LTA^-^ strains exhibited major differences in the levels of intracellular proteins identified from the SLAP fraction, indicating increased permeability or autolysis in the LTA-deficient strains causing liberation of intracellular components.

The impact of LTA on cell division has been investigated in a few species, with varying effects depending on growth phase and cell morphology. This can partially be attributed to the role of LTA in regulating autolysins through maintenance of ion homeostasis, but it is also known that LTA facilitates assembly or localization of the FtsZ ring during cell division (Schirner et al., 2009, Reichmann et al., 2014). An early report demonstrated that LTA binding of *N*-acetylmuramoyl-L-alanine amidase inhibited its activity, while the addition of cations restored activity (Bierbaum and Sahl, 1987). Additional studies reported that deletion of *ltaS* correlates with lower levels of peptidoglycan hydrolases in *S. aureus* (Oku et al., 2009) and substantiated direct association between LTA and autolysins in the cell wall (Steen et al., 2003). Collectively, these results indicate that LTA has the capacity to regulate autolysins directly through association or exclusion and indirectly through ion homeostasis. It has long been proposed that LTA functions in ion homeostasis via its negatively charged poly-glycerol/ribitol phosphate backbone causing proton and divalent cation sequestration (Heptinstall et al., 1970). Experimentally this was substantiated by growing *B. subtilis* and an LTA-deficient derivative under various concentrations of Mn^2+^, from which a sophisticated proposal of LTA-mediated ionic regulation emerged (Schirner et al., 2009). We also observed that LTA-deficient *L. acidophilus* was sensitive to an elevated Mn^2+^ concentration, suggesting that even in disparate Gram-positive bacteria with vastly different ionic growth requirements, LTA influences extracellular ionic milieu.

Large scale proteomic methods are being increasingly applied to probiotic and fermentative lactic acid bacteria, with particular focus on exoproteome profiling (Johnson et al., 2013, 2016). This is a promising approach to defining the molecular basis for probiotic activity since extracellular proteins appear to be strain-specific and are uniquely positioned to interact with the environment, whether it be a food or mucosal niche. Striking differences in the SLAP profiles of the LTA-deficient strains were apparent. Notably, the distribution of spectral counts for intracellular proteins without a predicted secretion signal was higher in both of the LTA-deficient strains, but was remarkable in NCK2025. It has been suggested that LTA acts as a barrier that reduces cell wall permeability, but it may also be possible that the upregulated peptidoglycan hydrolases can cause increased autolysis. Deletion of *ltaS* also impacted two glycerol-3-phosphate ABC transporters, which likely supply the phosphoglycerol transferase with glycerol-3-phosphate necessary for polymerization of the polyglycerolphosphate LTA backbone.

Our results indicate functions of LTA in cell division and cell morphology, and have underscored phenotypic results with compensatory transcriptional and exoproteomic changes. This approach has resulted in identification of genes that may be directly related to LTA activity on the cell surface. By determining the consequences of deleting a specific gene/subset of genes on global gene expression and exoproteome composition it is possible to uncover regulatory networks and cellular processes relevant to the genotype. These results underscore how deleting *ltaS*, a conserved pleiotropic gene, impacts the trancriptome and exoproteome of *L. acidophilus*, facilitating identification of genes influenced by LTA activity or relevant for its functions.

## Author Contributions

KS, primary author, designed research, performed research, primary contribution for organization and writing paper. YG, Ph.D. senior scientist, designed research, performed research, wrote paper, project advisor. BJ, performed research and wrote paper. SOF, Ph.D. senior scientist, designed research, performed research, wrote paper, project advisor. JMA, Ph.D. performed research and wrote paper. RB, senior advisor, designed research, edited paper. TK, corresponding author, designed research senior advisor, managed project, and edited paper.

## Conflict of Interest Statement

The authors declare that the research was conducted in the absence of any commercial or financial relationships that could be construed as a potential conflict of interest.

## Footnotes

1 http://www.bioinformatics.babraham.ac.uk/projects/fastqc/
