# Supplementary material for: Deletion of Lipoteichoic Acid Synthase Impacts Expression of Genes Encoding Cell Surface Proteins in Lactobacillus acidophilus
Source: Front Microbiol. 2017 Apr 11;8:553. doi: 10.3389/fmicb.2017.00553 (PMC5387067; doi:10.3389/fmicb.2017.00553)
Supplement: Supplementary file 1 [file Table_1.DOCX]

Supplemental Table 1. Primers

| Primers | Sequence (5’ to 3’) |
| --- | --- |
| Construction of Δ*slpB* and Δ*ltaS* | |
| slpB1U-F | GAAATAGGATCCCAGCTATCATCAGCCTTCAT |
| slpB2U-R | AGATACAGCAGAAGCAGCAA |
| slpB3D-F | TTGCTGCTTCTGCTGTATCTTTGAAGAAGGGTGAAGTTGT |
| slpB4D-R | TAAAGTAGAGCTCTGATAGGAAAGGTGCTCAAT |
| ltaS1U-F | CAGCAGGGATCCAAGGTGGTCGATCGACTTCTAT |
| ltaS2U-R | GGTACGTTCCATCGCTCTCT |
| ltaS3D-F | AGAGAGCGATGGAACGTACCACTGATGCGCCTGAGTTGAAA |
| ltaS4D-R | CTGCTGGAGCTCGCCCGCAGTAATCTTGACTT |
| PCR analysis and DNA sequencing of deletion targets | |
| slpB-up | TTCGTTGCATCAGCATAAG |
| slpB-dw | GTGTAGTATTGCCGATAACAG |
| ltaS-up | GTTCATGGCTTAGTGTTAC |
| ltaS-dw | CATCATCTGCTTCTTCATC |

^a^ restriction enzyme sites, underlined
