# Supplementary material for: Deletion of Lipoteichoic Acid Synthase Impacts Expression of Genes Encoding Cell Surface Proteins in Lactobacillus acidophilus
Source: Front Microbiol. 2017 Apr 11;8:553. doi: 10.3389/fmicb.2017.00553 (PMC5387067; doi:10.3389/fmicb.2017.00553)
Supplement: Supplementary file 2 [file Table_2.DOCX]

Supplemental Table 2. RNA-sequencing statistics.

| Strain | Total reads | Mapped Reads | Mean Coverage |
| --- | --- | --- | --- |
| NCK2025 | 19,318,068 | 19,097,780 | 1157.9 |
| NCK1909 | 17,846,697 | 17,806,574 | 1053.5 |
| NCK2030 | 20,113,787 | 15,482,388 | 1224.3 |
| NCK2187 | 18,530,899 | 18,434,744 | 1156.2 |
