# Supplementary material for: Deletion of Lipoteichoic Acid Synthase Impacts Expression of Genes Encoding Cell Surface Proteins in Lactobacillus acidophilus
Source: Front Microbiol. 2017 Apr 11;8:553. doi: 10.3389/fmicb.2017.00553 (PMC5387067; doi:10.3389/fmicb.2017.00553)
Supplement: Supplementary file 3 [file Table_3.DOCX]

Supplemental Table 3. Operons containing genes found to be differentially expressed in LTA-deficient strains.

| Operon | Putative function | Regulator-  effector | Gene | Range of log_2_ change |
| --- | --- | --- | --- | --- |
| LBA0444-LBA0447 | Lipoteichoic acid biosynthesis |  | LBA0444 | 0.3 to 0.4 |
|  |  |  | LBA0445 | 0.2 to 0.5 |
|  |  |  | LBA0446 | 0.5 to 0.7 |
|  |  |  | LBA0447* | -2.1 to -2.8 |
| LBA0541-LBA0544 | Heavy metal resistance | FlpA-Oxygen | LBA0541* | -1.3 to -1.8 |
|  |  |  | LBA0542* | -1.7 to -2.6 |
|  |  |  | LBA0543* | -1.7 to -2.5 |
|  |  |  | LBA0544* | -1.7 to -2.2 |
| LBA0852-LBA0857 | Lysine biosynthesis | RNA-Lysine | LBA0852 | -0.8 to -1.9 |
|  |  |  | LBA0853* | -1.1 to -1.7 |
|  |  |  | LBA0854 | -0.9 to -1.5 |
|  |  |  | LBA0855 | -0.3 to -1.5 |
|  |  |  | LBA0856 | -1.4 to 0.0 |
|  |  |  | LBA0857 | -1.1 to 0.2 |
| LBA1042-LBA1046 | Glutamine transport |  | LBA1042 | 0.2 to 1.2 |
|  |  |  | LBA1044 | 0.8 to 1.8 |
|  |  |  | LBA1045* | 1.1 to 2.0 |
|  |  |  | LBA1046 | 0.8 to 1.6 |
| LBA1184-LBA1189 | Antimicrobial export | YhcF | LBA1184* | 1.1 to 1.7 |
|  |  |  | LBA1186 | 0.8 to 1.3 |
|  |  |  | LBA1187 | 0.7 to 1.5 |
|  |  |  | LBA1188 | 0.8 to 1.5 |
|  |  |  | LBA1189 | 0.5 to 1.4 |
| LBA1679-LBA1680 | Antimicrobial export | YhcF | LBA1679* | 1.7 to 2.4 |
|  |  |  | LBA1680* | 1.7 to 2.8 |
